# Modifying effect of metabotype on diet–diabetes associations

**DOI:** 10.1007/s00394-019-01988-5

**Published:** 2019-05-14

**Authors:** Anna Riedl, Nina Wawro, Christian Gieger, Christa Meisinger, Annette Peters, Wolfgang Rathmann, Wolfgang Koenig, Konstantin Strauch, Anne S. Quante, Barbara Thorand, Cornelia Huth, Hannelore Daniel, Hans Hauner, Jakob Linseisen

**Affiliations:** 1 Independent Research Group Clinical Epidemiology, Helmholtz Zentrum München, German Research Center for Environmental Health (GmbH), Ingolstädter Landstr. 1, 85764 Neuherberg, Germany; 2 Chair of Epidemiology, Ludwig-Maximilians-Universität München, at UNIKA-T (Universitäres Zentrum für Gesundheitswissenschaften am Klinikum Augsburg), Neusässer Str. 47, 86156 Augsburg, Germany; 3 German Center for Diabetes Research (DZD e.V.), Ingolstädter Landstr. 1, 85764 Neuherberg, Germany; 4 Institute of Epidemiology, Helmholtz Zentrum München, German Research Center for Environmental Health (GmbH), Ingolstädter Landstr. 1, 85764 Neuherberg, Germany; 5 Research Unit of Molecular Epidemiology, Helmholtz Zentrum München, German Research Center for Environmental Health (GmbH), Ingolstädter Landstr. 1, 85764 Neuherberg, Germany; 6 Institute for Biometrics and Epidemiology, German Diabetes Center, Leibniz Center for Diabetes Research at Heinrich Heine University Düsseldorf, Auf’m Hennekamp 65, 40225 Düsseldorf, Germany; 7 DZHK (German Centre for Cardiovascular Research), Partner Site Munich Heart Alliance, Pettenkoferstr. 8a and 9, 80336 Munich, Germany; 8 Deutsches Herzzentrum München, Technische Universität München, Lazarettstr. 36, 80636 Munich, Germany; 9 Department of Internal Medicine II-Cardiology, University of Ulm Medical Center, Albert-Einstein-Allee 23, 89081 Ulm, Germany; 10 Institute of Genetic Epidemiology, Helmholtz Zentrum München, German Research Center for Environmental Health (GmbH), Ingolstädter Landstr. 1, 85764 Neuherberg, Germany; 11 Chair of Genetic Epidemiology, IBE, Faculty of Medicine, Ludwig-Maximilians-Universität München, Marchioninistr. 15, 81377 Munich, Germany; 12 Department of Gynecology and Obstetrics, University Hospital rechts der Isar, Technical University Munich, Ismaninger Str. 22, 81675 Munich, Germany; 13 Chair of Nutritional Physiology, Technical University of Munich, Gregor-Mendel-Str. 2, 85354 Freising-Weihenstephan, Germany; 14 Else Kröner-Fresenius Centre for Nutritional Medicine, Technical University of Munich, Gregor-Mendel-Str. 2, 85354 Freising-Weihenstephan, Germany; 15 ZIEL, Institute for Food and Health, Technical University of Munich, Weihenstephaner Berg 1, 85354 Freising-Weihenstephan, Germany; 16 Institute of Nutritional Medicine, University Hospital rechts der Isar, Technical University of Munich, Uptown München Campus D, Georg-Brauchle-Ring 60/62, 80992 Munich, Germany

**Keywords:** Diabetes, Diet, *enable*-Cluster, Metabolic phenotype, Metabotype

## Abstract

**Purpose:**

Inter-individual metabolic differences may be a reason for previously inconsistent results in diet–diabetes associations. We aimed to investigate associations between dietary intake and diabetes for metabolically homogeneous subgroups (‘metabotypes’) in a large cross-sectional study.

**Methods:**

We used data of 1517 adults aged 38–87 years from the German population-based KORA FF4 study (2013/2014). Dietary intake was estimated based on the combination of a food frequency questionnaire and multiple 24-h food lists. Glucose tolerance status was classified based on an oral glucose tolerance test in participants without a previous diabetes diagnosis using American Diabetes Association criteria. Logistic regression was applied to examine the associations between dietary intake and diabetes for two distinct metabotypes, which were identified based on 16 biochemical and anthropometric parameters.

**Results:**

A low intake of fruits and a high intake of total meat, processed meat and sugar-sweetened beverages (SSB) were significantly associated with diabetes in the total study population. Stratified by metabotype, associations with diabetes remained significant for intake of total meat (OR 1.67, 95% CI 1.04–2.67) and processed meat (OR 2.23, 95% CI 1.24–4.04) in the metabotypes with rather favorable metabolic characteristics, and for intake of fruits (OR 0.83, 95% CI 0.68–0.99) and SSB (OR:1.21, 95% CI 1.09–1.35) in the more unfavorable metabotype. However, only the association between SSB intake and diabetes differed significantly by metabotype (*p* value for interaction = 0.01).

**Conclusions:**

Our findings suggest an influence of metabolic characteristics on diet–diabetes associations, which may help to explain inconsistent previous results. The causality of the observed associations needs to be confirmed in prospective and intervention studies.

**Electronic supplementary material:**

The online version of this article (10.1007/s00394-019-01988-5) contains supplementary material, which is available to authorized users.

## Introduction

Type 2 diabetes mellitus (T2DM) with its adverse health consequences for individuals and its financial burden on healthcare systems is an important public health issue worldwide [1, 2]. In Germany, the prevalence of known T2DM was 8.5% in 2009 and 9.5% in 2015, and it is expected to rise further due to an ageing population with an increase in unhealthy lifestyle [1, 3]. It has to be assumed that the actual prevalence of T2DM is even higher due to a large number of undiagnosed individuals [4, 5].

Changes in lifestyle, for example in dietary behavior, may prevent or delay the development of T2DM [6–8]. However, previous studies investigating the impact of food on the risk of T2DM have shown inconsistent results [9, 10].

It is well established that the variability of metabolic characteristics between individuals leads to differences in the response to dietary factors [11–15]. This could be a reason why associations of food groups and nutrients with T2DM are often weak or even different between studies. The identification of metabolically homogeneous subgroups of the population, so-called metabotypes or metabolic phenotypes [16–21], has already been performed several times [16, 22, 23], and may help to better understand the inconsistency in diet-T2DM associations across studies. Furthermore, this may be relevant in diabetes prevention for the development of targeted dietary recommendations at the metabotype subgroup level, which may be more effective than general dietary advice [11, 14, 16, 24, 25].

Therefore, we aimed (1) to identify distinct metabotypes and (2) to investigate the cross-sectional associations of intake of several food groups and nutrients with T2DM stratified by metabotype subgroup in the large population-based Cooperative Health Research in the Region of Augsburg (KORA) FF4 study.

## Methods

### Study population

Analyses were performed on data from the population-based KORA FF4 (2013/2014) study, the second follow-up of the KORA S4 health survey conducted in the region of Augsburg in Southern Germany between 1999 and 2001 [26]. In brief, of the 4261 participants included in S4, 2279 individuals also participated in the 14-year follow-up FF4 study. Detailed information on the participation response has been given elsewhere [27]. All individuals answered self-administered questionnaires, and participated in a standardized physical examination as well as in a computer-assisted face-to-face interview conducted by trained investigators at the study center. A detailed description has been provided previously [5].

### Ethical standards

All participants gave their written informed consent, and the study was approved by the Ethics Committee of the Bavarian Chamber of Physicians and conducted in accordance with the Declaration of Helsinki.

### Assessment of glucose tolerance status

Prevalent diabetes was defined by either current intake of antidiabetic medication or a self-reported diagnosis, both validated with the respective treating physician. All participants without previously known diabetes took part in a standard oral glucose tolerance test (OGTT) and their glucose tolerance status was classified according to the 2003 American Diabetes Association (ADA) diagnostic criteria [28]. Further details have been outlined elsewhere [29]. An OGTT value of ≥ 7.0 mmol/L fasting or ≥ 11.1 mmol/L 2-h glucose was defined as undetected diabetes mellitus (UDM), also called screen-detected diabetes. Participants with isolated impaired fasting glucose (IFG 5.6–6.9 mmol/L fasting glucose), isolated impaired glucose tolerance (IGT 7.8–11.0 mmol/L 2-h glucose) or combined IFG/IGT were classified as prediabetic. Individuals with fasting glucose levels < 5.6 mmol/L and 2-h glucose levels < 7.8 mmol/L were classified as normal glucose tolerant (NGT).

### Assessment of dietary intake

Dietary intake was assessed in 1602 KORA FF4 participants with up to three 24-h food lists [30] and a food frequency questionnaire. Combining this information, the usual dietary intake was estimated in an advanced blended two-step approach, which follows the idea of the National Cancer Institute (NCI) method and the Multiple Source Method (MSM) [31, 32] to separate the calculation of consumption amount and consumption probability. The consumption probability and the consumption amount on consumption days were estimated separately with models both including the same covariates to link the two parts. Then, the usual dietary intake of all food items was calculated for each participant by multiplying the consumption probability of a certain food item by the usual consumption amount on a consumption day. The food groups were categorized according to the European Prospective Investigation into Cancer and Nutrition (EPIC)-Soft classification system [33] and nutrients were derived using the National Nutrient Database (Bundeslebensmittelschlüssel BLS 3.02). For the analysis, we selected the 17 following food groups and nutrients in g/d associated with T2DM in the literature [9, 10, 34, 35]: fruits, vegetables, potatoes, total meat, red meat (beef and pork), poultry, processed meat, eggs, total dairy, milk, yogurt, cheese, coffee, fruit and vegetable juice, sugar-sweetened beverages (SSB), alcohol and fiber.

### Assessment of covariates

The selection of covariates was based on theoretical considerations and the existing literature on diet and diabetes [9]. These included age (years), sex (reference = male), energy intake (kcal/day), waist circumference (cm), family history of diabetes [yes, no (= reference), do not know], physical activity [active in summer and in winter and active for ≥ 1 h per week in at least one season, inactive (= reference)], smoking status [never (= reference), former, current], hypertension [≥ 140/90 mmHg or antihypertensive medication given that the participants were aware of having hypertension; yes, no (= reference)] and education [< 10 years (= reference), 10 to < 13 years, ≥ 13 years, in accordance with the German education system]. Waist circumference and blood pressure were measured at the study center under standardized conditions by trained staff. All other covariates were assessed during a standardized personalized computer-assisted interview or via a self-administered questionnaire.

### Statistical analysis

We performed all statistical analyses using the statistical software package RStudio version 1.0.136 that uses R version 3.2.2 (R Development Core Team, 2010, http://www.r-project.org). *p* values of < 0.05 were considered statistically significant.

#### Identification of metabotypes

The metabotypes were identified in KORA FF4 analogous to Riedl et al. [23] in KORA F4. Of the 34 originally used anthropometric and fasting biochemical blood parameters in KORA F4, a subset of 16 parameters was also available in KORA FF4 for the definition of metabotypes. These included body mass index (BMI), and the following blood biomarkers: glucose, total cholesterol, high density lipoprotein cholesterol, total cholesterol/high density lipoprotein cholesterol ratio, low-density lipoprotein cholesterol, glycated hemoglobin, uric acid, triglycerides, leukocytes, gamma-glutamyltransferase, glutamate-pyruvate transaminase, glutamate–oxaloacetate transaminase, alkaline phosphatase, high-sensitivity C-reactive protein and insulin.

In the preprocessing step, the KORA FF4 study population (*n* = 2279) was reduced to 2218 participants by excluding 54 participants who did not fast for at least 8 h before blood collection and by excluding 7 participants with more than 10% missing values of all clustering variables listed above. The remaining missing values in the clustering variables were imputed using the R package “mice” (multivariate imputation by chained equations) version 2.25 [36] generating five complete data sets with ten iterations each. All biochemical and anthropometric parameters were *z*-standardized prior to clustering. Subsequently, the 2218 FF4 participants were divided into three clusters using the k-means cluster algorithm of the R package “miclust” (multiple imputation in cluster analysis) version 1.2.5 [37] based on all 16 biochemical and anthropometric parameters available in this study. A detailed metabolic characterization of these clusters representing metabotypes is provided in the Online Resource (Supplemental Table 1). Further details on the identification procedure of metabotypes are given elsewhere [23].

#### Analysis of associations between dietary intake and diabetes

Of the total 2218 FF4 participants with metabotype information, those with type 1 diabetes mellitus (*n* = 4), unclear glucose tolerance status (*n* = 67) or missing information on dietary intake (*n* = 628) or covariate data (*n* = 2) were excluded from the analyses resulting in a final sample size of 1517 participants. All food groups were rescaled for 50 g/day increments. Among the investigated nutrients, fiber was rescaled to 10 g/day and alcohol intake was classified by sex in accordance with the reference values of the German Nutrition Society (Deutsche Gesellschaft für Ernährung) as low (< 5 g/day for men and < 2 g/day for women), moderate (5 to < 20 g/day for men and 2 to < 10 g/day for women) or high (≥ 20 g/day for men and ≥ 10 g/day for women) [38]. Due to the low diabetes prevalence in metabotype clusters 1 and 2 (see Table 1), these groups were combined and analyzed together in comparison to cluster 3.

**Table 1:** Demographic baseline characteristics of the total study population and across the three metabotype clusters, KORA FF4 study

	Total	Metabotypes		
		Cluster 1	Cluster 2	Cluster 3
	*N* = 1517	*N* = 678	*N* = 539	*N* = 300
Glucose tolerance status				
NGT	777 (51.2)	485 (71.5)	254 (47.1)	38 (12.7)
Prediabetes	539 (35.5)	160 (23.6)	244 (45.3)	135 (45.0)
UDM	64 (4.2)	12 (1.8)	23 (4.3)	29 (9.7)
Prevalent T2DM	137 (9.0)	21 (3.1)	18 (3.3)	98 (32.7)
Sex				
Men	743 (49.0)	228 (33.6)	313 (58.1)	202 (67.3)
Women	774 (51.0)	450 (66.4)	226 (41.9)	98 (32.7)
Age (years)				
Median (25th, 75th)	60.0 (50.0, 69.0)	55.0 (47.0, 66.0)	61.0 (52.0, 69.0)	65.0 (56.8, 73.0)
Education (years)				
< 10	76 (5.0)	25 (3.7)	33 (6.1)	18 (6.0)
10 to < 13	885 (58.3)	365 (53.8)	325 (60.3)	195 (65.0)
≥ 13	556 (36.7)	288 (42.5)	181 (33.6)	87 (29.0)
BMI (kg m^−2^)				
Median (25th, 75th)	26.9 (24.3, 30.3)	24.6 (22.7, 26.8)	27.8 (25.6, 30.4)	31.4 (28.6, 35.4)
Underweight	6 (0.4)	6 (0.9)	0 (0.0)	0 (0.0)
Normal	483 (31.8)	361 (53.2)	103 (19.1)	19 (6.3)
Overweight	627 (41.3)	252 (37.2)	281 (52.1)	94 (31.3)
Obese	401 (26.4)	59 (8.7)	155 (28.8)	187 (62.3)
Waist circumference (cm)				
Median (25th, 75th)	96.3 (85.7, 105.4)	86.9 (79.1, 96.1)	98.9 (92.3, 105.3)	109.7 (102.3, 117.4)
Physical activity				
Inactive	587 (38.7)	209 (30.8)	205 (38.0)	173 (57.7)
Active	930 (61.3)	469 (69.2)	334 (62.0)	127 (42.3)
Smoking status				
Nonsmoker	637 (42.0)	304 (44.8)	224 (41.6)	109 (36.3)
Ex-smoker	675 (44.5)	281 (41.4)	237 (44.0)	157 (52.3)
Smoker	205 (13.5)	93 (13.7)	78 (14.5)	34 (11.3)
Family history of diabetes				
Yes	496 (32.7)	200 (29.5)	179 (33.2)	117 (39.0)
No	879 (57.9)	435 (64.2)	302 (56.0)	142 (47.3)
Do not know	142 (9.4)	43 (6.3)	58 (10.8)	41 (13.7)
Hypertension				
Yes	588 (38.8)	174 (25.7)	210 (39.0)	204 (68.0)
No	929 (61.2)	504 (74.3)	329 (61.0)	96 (32.0)

Median (25th, 75th percentile) for continuous variables and *n* (column %) for categorical variables

*BMI* body mass index, *KORA* Cooperative Health Research in the Region of Augsburg, *NGT* normal glucose tolerance, *T2DM* type 2 diabetes mellitus, *UDM* undetected diabetes mellitus

To examine the cross-sectional associations between dietary intake and diabetes dichotomized in NGT/prediabetes (= reference) and UDM/prevalent T2DM, binary logistic regression was performed. For each of the dietary intake variables, two models with different sets of covariates were fitted: the basic model was adjusted for age, sex and energy intake; the fully adjusted model was additionally adjusted for waist circumference, family history of diabetes, physical activity, smoking, education, hypertension, and metabotype. Thus, the respective models differed only in the dietary intake variable used, but included the same sample size and covariates. All analyses were performed for the total study population and stratified by metabotype subgroup.

Likelihood ratio tests were used to detect possible interaction effects between metabotypes and the respective dietary intake variables in the fully adjusted model. Significant results indicated differences in diet–diabetes associations between metabotype subgroups. A flow chart showing the overall analysis strategy is provided in the Online Resource (Supplemental Fig. 1).

As a sensitivity analysis, we fitted intermediate adjusted models removing the covariates hypertension and waist circumference from the fully adjusted models, as these are rather intermediary/mediating variables than real confounders in diet–diabetes associations. In another sensitivity analysis, we restricted the study population to adults aged ≥ 60 years to investigate age-specific effects.

## Results

The number of individuals at each stage of our analysis is shown in the flow chart in Supplemental Fig. 1 provided in the Online Resource, leaving a final study population of 1517 individuals for the analysis of diet–diabetes associations.

Table 1 presents the characteristics of the total study population and for each of the three metabotypes identified in KORA FF4. The total study population consists of approximately equal proportions of men and women with an age range of 38–87 years. Of 1517 participants, 777 (51.2%) had NGT, 539 (35.5%) had prediabetes, 64 (4.2%) had UDM and 137 (9.0%) had prevalent T2DM. By metabotyping, 678 participants (about 45%) were grouped into cluster 1, 539 participants (about 36%) into cluster 2, and 300 participants (about 20%) into cluster 3. The proportion of participants with UDM and prevalent T2DM was higher in cluster 3 compared to cluster 2 and cluster 1, respectively (UDM: 9.7% vs. 4.3% vs. 1.8%; prevalent T2DM: 32.7% vs. 3.3% vs. 3.1%). Cluster 3 showed the highest proportions of men, individuals with a positive family history of diabetes and hypertension. At the same time, cluster 3 showed the lowest percentages of highly educated and physically active participants as well as current smokers. Participants of cluster 3 were further characterized by the highest median age, BMI and waist circumference.

Table 2 shows the median usual dietary intake of participants in the combined clusters 1 and 2 and of participants in cluster 3, in total and according to glucose tolerance status (NGT/prediabetes and UDM/prevalent T2DM). The intake of total meat, red meat, processed meat, SSB and total energy was higher and the intake of vegetables, total dairy, milk, yogurt and fruit and vegetable juice was lower in cluster 3 compared to the combined clusters 1 and 2. In both metabotype subgroups, the intake of potatoes and coffee was higher and the intake of milk, fruit and vegetable juice and SSB was lower in individuals with UDM/prevalent T2DM compared to individuals with NGT/prediabetes. Participants with UDM/prevalent T2DM in clusters 1 and 2 showed a higher intake of total meat and total energy, whereas participants with UDM/prevalent T2DM in cluster 3 showed a lower intake of total meat and total energy compared to the participants with NGT/prediabetes of the respective metabotype subgroup.

**Table 2:** Usual dietary intake of the study population by metabotype subgroup for NGT/prediabetes and UDM/prevalent T2DM, KORA FF4 study

	Total	NGT/prediabetes	UDM/prevalent T2DM
Total	*N* = 1517	*N* = 1316	*N* = 201
Cluster 1/cluster 2	*N* = 1217	*N* = 1143	*N* = 74
Cluster 3	*N* = 300	*N* = 173	*N* = 127

	Median (25%, 75%)	Median (25%, 75%)	Median (25%, 75%)
Food item			
Fruits (g/day)			
Cluster 1/cluster 2	145 (92, 206)	145 (91, 205)	146 (122, 215)
Cluster 3	148 (90, 214)	147 (89, 209)	149 (96, 214)
Vegetables (g/day)			
Cluster 1/cluster 2	168 (137, 210)	168 (137, 211)	165 (132, 195)
Cluster 3	149 (121, 184)	149 (122, 184)	147 (121, 185)
Potatoes (g/day)			
Cluster 1/cluster 2	55 (44, 70)	54 (44, 69)	64 (53, 84)
Cluster 3	59 (48, 78)	57 (46, 75)	63 (50, 82)
Total meat (g/day)			
Cluster 1/cluster 2	102 (81, 134)	102 (80, 133)	115 (88, 141)
Cluster 3	136 (102, 161)	140 (104, 167)	133 (102, 160)
Red meat (g/day)			
Cluster 1/cluster 2	25 (19, 33)	25 (19, 33)	28 (19, 36)
Cluster 3	30 (22, 40)	29 (23, 40)	30 (22, 40)
Poultry (g/day)			
Cluster 1/cluster 2	13 (10, 18)	13 (10, 18)	11 (11, 17)
Cluster 3	13 (11, 19)	13 (11, 19)	13 (10, 19)
Processed meat (g/day)			
Cluster 1/cluster 2	39 (28, 57)	38 (28, 56)	50 (33, 69)
Cluster 3	59 (42, 81)	58 (42, 81)	61 (45, 80)
Eggs (g/day)			
Cluster 1/cluster 2	11 (8, 17)	11 (8, 16)	12 (10, 22)
Cluster 3	13 (9, 20)	12 (8, 20)	14 (9, 20)
Total dairy (g/day)			
Cluster 1/cluster 2	186 (122, 264)	188 (125, 267)	162 (105, 212)
Cluster 3	149 (94, 224)	148 (91, 226)	150 (95, 218)
Milk (g/day)			
Cluster 1/cluster 2	77 (30, 138)	78 (30, 140)	53 (32, 115)
Cluster 3	47 (16, 100)	52 (16, 105)	44 (16, 87)
Yogurt (g/day)			
Cluster 1/cluster 2	32 (14, 70)	33 (14, 71)	24 (13, 51)
Cluster 3	23 (13, 56)	23 (13, 51)	23 (13, 56)
Cheese (g/day)			
Cluster 1/cluster 2	27 (19, 37)	27 (19, 37)	28 (20, 38)
Cluster 3	26 (19, 37)	26 (20, 37)	26 (18, 36)
Coffee (g/day)			
Cluster 1/cluster 2	435 (366, 479)	433 (366, 478)	449 (349, 501)
Cluster 3	438 (331, 483)	420 (303, 476)	456 (374, 499)
Fruit and vegetable juice (g/day)			
Cluster 1/cluster 2	45 (24, 115)	45 (24, 116)	32 (22, 86)
Cluster 3	36 (24, 125)	43 (26, 167)	30 (23, 108)
SSB (g/day)			
Cluster 1/cluster 2	6 (3, 16)	6 (3, 17)	5 (3, 9)
Cluster 3	10 (5, 58)	11 (6, 47)	7 (4, 66)
Nutrient			
Energy intake (kJ/day)			
Cluster 1/cluster 2	7657 (6520, 8872)	7615 (6509, 8885)	7972 (6595, 8772)
Cluster 3	7821 (6516, 9136)	8007 (6657, 9296)	7672 (6334, 8810)
Alcohol (g/day)			
Cluster 1/cluster 2	5 (3, 14)	5 (3, 14)	5 (2, 16)
Cluster 3	6 (3, 17)	7 (3, 19)	5 (2, 14)
Total fiber (g/day)			
Cluster 1/cluster 2	18 (14, 21)	17 (14, 21)	18 (16, 21)
Cluster 3	16 (14, 19)	16 (14, 19)	16 (14, 19)

Median (25th, 75th percentile)

*KORA* Cooperative Health Research in the Region of Augsburg, *NGT* normal glucose tolerance, *SSB* sugar-sweetened beverages, *T2DM* type 2 diabetes mellitus, *UDM* undetected diabetes mellitus

In the total study population, intake of fruits and fiber as well as moderate alcohol consumption showed negative associations and intake of total meat, red meat, processed meat and SSB showed positive associations with UDM/prevalent T2DM (all *p* < 0.05) in the basic model. These results are provided in Supplemental Table 2 in the Online Resource. Table 3 displays the odds ratios (OR) and 95% confidence intervals (CI) of the fully adjusted logistic regression models for the total study population. After adjustment for the additional covariates, associations remained significant for intake of fruits (OR per increase in intake amount by 50 g/day: 0.86, 95% CI 0.75–0.98), total meat (OR 1.50, 95% CI 1.09–2.08), processed meat (OR 1.83, 95% CI 1.22–2.77) and SSB (OR 1.09, 95% CI 1.01–1.17) (all *p* < 0.05).

**Table 3:** Fully adjusted associations between the consumption of various food items and nutrients with UDM/Prevalent T2DM in the total study population, KORA FF4 study

Food or nutrient	Fully adjusted model	
	OR	95% CI
Fruits (50 g/day)	**0.86**	**0.75–0.98**
Vegetables (50 g/day)	1.17	0.96–1.43
Potatoes (50 g/day)	1.16	0.73–1.84
Total meat (50 g/day)	**1.50**	**1.09–2.08**
Red meat (50 g/day)	1.01	0.39–2.53
Poultry (50 g/day)	1.50	0.38–5.51
Processed meat (50 g/day)	**1.83**	**1.22–2.77**
Eggs (50 g/day)	0.85	0.39–1.75
Total dairy (50 g/day)	1.00	0.90–1.11
Milk (50 g/day)	0.97	0.85–1.10
Yogurt (50 g/day)	1.08	0.86–1.33
Cheese (50 g/day)	1.58	0.76–3.27
Coffee (50 g/day)	1.02	0.96–1.09
Fruit and vegetable juice (50 g/day)	0.97	0.87–1.07
SSB (50 g/day)	**1.09**	**1.01–1.17**
Moderate alcohol consumption^a^	1.03	0.67–1.57
High alcohol consumption^a^	0.89	0.52–1.52
Total fiber (10 g/day)	1.11	0.62–1.93

Logistic regression models: reference category = NGT/prediabetes. Fully adjusted models adjusted for age, sex, energy intake, waist circumference, family history of diabetes, physical activity, smoking, education, hypertension and metabotype. Significant results (*p* < 0.05) printed in bold

*N* = 1517

*CI* confidence interval, *KORA* Cooperative Health Research in the Region of Augsburg, *NGT* normal glucose tolerance, *OR* odds ratio, *SSB* sugar-sweetened beverages, *T2DM* type 2 diabetes mellitus, *UDM* undetected diabetes mellitus

^a^Compared against low alcohol intake (< 5 g/day for men, < 2 g/day for women as reference category); moderate considered 5 to < 20 g/day for men, 2 to < 10 g/day for women; high considered ≥ 20 g/day for men, ≥ 10 g/day for women

The results of the fully adjusted models stratified by metabotype subgroup are presented in Table 4 (results of the basic model are shown in Supplemental Table 3 in the Online Resource). The positive associations of intake of total meat (OR 1.67, 95% CI 1.04–2.67) and processed meat (OR 2.23, 95% CI 1.24–4.04) with UDM/prevalent T2DM, per increase in intake amount by 50 g/day, remained significant in the combined clusters 1 and 2 only. In contrast, the negative association of intake of fruits (OR 0.83, 95% CI 0.68–0.99) and the positive association of intake of SSB (OR 1.21, 95% CI 1.09–1.35) with UDM/prevalent T2DM, per increase in intake amount by 50 g/day, remained significant in cluster 3 only. Significant interaction effects with metabotype subgroups were determined for intake of eggs (*p* = 0.02) and SSB (*p* = 0.01). Analyses were not further stratified by sex due to non-significance of interaction effects (*p* ≥ 0.05) with dietary intake.

**Table 4:** Fully adjusted associations between the consumption of various food items and nutrients with UDM/prevalent T2DM stratified by metabotype subgroup, KORA FF4 study

Food or nutrient	Cluster 1/cluster 2 *N* = 1217		Cluster 3 *N* = 300		*p* value interaction^b^
	OR	95% CI	OR	95% CI	
Fruits (50 g/day)	0.91	0.75–1.11	**0.83**	**0.68**–**0.99**	0.24
Vegetables (50 g/day)	1.25	0.94–1.64	1.09	0.80–1.47	0.63
Potatoes (50 g/day)	0.82	0.41–1.57	1.59	0.80–3.23	0.74
Total meat (50 g/day)	**1.67**	**1.04–2.67**	1.51	0.95–2.41	0.16
Red meat (50 g/day)	0.95	0.20–3.92	0.89	0.26–3.03	0.49
Poultry (50 g/day)	0.79	0.07–6.81	2.29	0.40–13.42	0.64
Processed meat (50 g/day)	**2.23**	**1.24**–**4.04**	1.79	1.00–3.26	0.11
Eggs (50 g/day)	1.82	0.65–4.73	0.38	0.12–1.14	**0.02**
Total dairy (50 g/day)	0.97	0.84–1.12	1.02	0.89–1.18	0.83
Milk (50 g/day)	0.93	0.76–1.10	1.00	0.83–1.21	0.70
Yogurt (50 g/day)	1.05	0.75–1.41	1.14	0.83–1.59	0.71
Cheese (50 g/day)	2.44	0.87–6.67	1.02	0.36–2.91	0.13
Coffee (50 g/day)	0.97	0.88–1.07	1.08	0.98–1.18	0.09
Fruit and vegetable juice (50 g/day)	0.98	0.83–1.13	0.97	0.84–1.12	0.53
SSB (50 g/day)	0.92	0.73–1.08	**1.21**	**1.09**–**1.35**	**0.01**
Moderate alcohol consumption^a^	0.97	0.53–1.76	1.05	0.55–1.98	0.75
High alcohol consumption^a^	0.84	0.39–1.78	0.81	0.37–1.77	
Total fiber (10 g/day)	1.44	0.65–3.08	0.87	0.36–2.09	0.29

Logistic regression models: reference category = NGT/prediabetes. Fully adjusted models adjusted for age, sex, energy intake, waist circumference, family history of diabetes, physical activity, smoking, education and hypertension. Significant results (*p* < 0.05) printed in bold

*N* = 1517

*CI* confidence interval, *KORA* Cooperative Health Research in the Region of Augsburg, *NGT* normal glucose tolerance, *OR* odds ratio, *SSB* sugar-sweetened beverages, *T2DM* type 2 diabetes mellitus, *UDM* undetected diabetes mellitus

^a^Compared against low alcohol intake (< 5 g/day for men, < 2 g/day for women as reference category); moderate considered 5 to < 20 g/day for men, 2 to < 10 g/day for women; high considered ≥ 20 g/day for men, ≥ 10 g/day for women

^b^*p* value of likelihood ratio test for the comparison of models with and without the interaction term of metabotype and the respective food or nutrient

The results (ORs, 95% CIs and *p* values for interaction) of the sensitivity analysis with intermediate adjusted models, that means without the covariates hypertension and waist circumference, are shown in Supplemental Table 4 and Supplemental Table 5 in the Online Resource. These have not changed significantly in the total study population. In the analyses stratified by metabotype, the results were also only slightly different. However, the associations of intake of total meat and processed meat with UDM/prevalent T2DM in cluster 3 reached statistical significance. Likewise, the sensitivity analysis restricted to 762 adults aged ≥ 60 years showed results similar to the analyses of the total study population (data not shown).

## Discussion

### Summary of main results

In the KORA FF4 study population, a low intake of fruits and a high intake of total meat, processed meat and SSB were significantly associated with UDM/prevalent T2DM. Taking into account metabolic differences between individuals, each of these associations remained significant only in one of both metabotype subgroups. In the combined clusters 1 and 2, the intake of total meat and processed meat showed a positive association with UDM/prevalent T2DM. In cluster 3, the intake of fruits was negative and the intake of SSB was positively associated with UDM/prevalent T2DM. Despite these differences in significant associations between metabotype subgroups, only the interaction effect between intake of SSB and metabotype was significant. In addition, the association between the intake of eggs and UDM/prevalent T2DM was also significantly different between metabotype subgroups, however, egg intake was not significantly associated with UDM/prevalent T2DM in the individual metabotype subgroups potentially caused by loss of statistical power due to stratification. Thus, further significant associations or significant differences in diet–diabetes associations between metabotypes as for example for intake of coffee (*p* value for interaction = 0.09) and processed meat (*p* value for interaction = 0.11) might be detectable in larger cohorts.

### Discussion of results regarding the existing literature on diet–diabetes associations

In general, the associations of intakes of fruits, total meat, processed meat and SSB with UDM/prevalent T2DM that we found in the total KORA FF4 study population are in line with previous meta-analyses and review articles on the respective food groups [39–48]. However, results of a number of other studies were inconsistent [9, 10], and there are studies that have shown either no or only weak associations between these food groups and diabetes [49–57]. All other selected dietary intake variables were not associated with UDM/prevalent T2DM in our study, in contrast to some of the previous studies [9, 10, 34, 35]. Our study was the first to investigate associations between diet and diabetes stratifying by metabotype, i.e., considering metabolic differences between individuals. As we identified intakes of fruits, total meat, processed meat and SSB to be significantly associated with diabetes in only one of both metabotypes, metabolic differences may partially explain conflicting results in diet–diabetes associations observed in previous studies. This holds mainly for the association between intake of SSB and diabetes, which was shown to be significantly different between both metabotype subgroups. Despite previously described age-related metabolic differences [58], the results of our sensitivity analysis restricted to older participants ≥ 60 years remained relatively stable. Consequently, these metabotypes may be relevant for adult populations with a large age range and not only for adult populations with specific age categories.

### Metabotypes for the development of targeted dietary recommendations for diabetes prevention

In the previous literature, there are numerous studies on metabotyping [16, 22, 23]. Some of these studies defined metabotypes including fasting plasma values, while a few studies classified metabotypes based on plasma parameter responsiveness to dietary interventions [17, 22, 59–62]. Fewer studies tested metabotype subgroups for a differential responsiveness to dietary intervention in a disease-specific manner [63–65]. For example, O’Sullivan et al. [63] identified a vitamin D-responsive metabotype subgroup concerning markers of the metabolic syndrome, Moazzami et al. [64] found subgroups of individuals with different insulin response after an intervention with breads, and Vázquez-Fresno [65] detected a responsive metabotype subgroup of cardiovascular risk patients to red wine polyphenols. In addition, O’Donovan et al. were the only ones developing dietary recommendations based on metabotype subgroups which was done using a decision tree approach [66, 67]. Thus to date, metabotypes have been rarely used for the development and establishment of targeted dietary recommendations for disease prevention. Further research should identify discrete differences between metabotype subgroups in the context of diet–disease relationships. By assigning individuals to metabotype subgroups, targeted dietary recommendations in disease prevention may be implemented in whole populations.

Concerning the metabotypes identified in KORA FF4, diabetes prevention may be especially relevant for cluster 3, defining an unfavorable metabotype concerning metabolic characteristics. In detail, this cluster showed the highest median concentrations of glucose and glycated hemoglobin, which are used in the diagnosis of prediabetes and prevalent T2DM [68]. Consequently, there were high numbers of individuals with prediabetes, UDM and prevalent T2DM in this cluster. However, clusters 1 and 2 defining a rather beneficial metabotype could also benefit from targeted prevention due to the high prevalence of prediabetes, which is a strong risk factor for the development of T2DM [69]. Other known risk factors for diabetes such as age, obesity, physical inactivity, family history of diabetes and hypertension were most frequent in cluster 3 [1, 68, 70]. In addition, low education as seen in cluster 3 was linked to poor health [71]. The lowest percentage of current smokers and simultaneous highest percentage of ex-smokers in cluster 3 indicated smoking cessation due to the high diabetes prevalence in this cluster, as smoking is as well a strong risk factor for diabetes [1, 70]. When assessing dietary intake in our metabotype subgroups, individuals in cluster 3 showed a higher intake of total energy, total meat, red meat, processed meat and SSB than individuals in clusters 1 and 2. Simultaneously, individuals in cluster 3 consumed lower amounts of vegetables, total dairy, milk, yogurt and fruit and vegetable juice compared to individuals in clusters 1 and 2. This dietary pattern was shown to be associated with increased risk for diabetes [9, 10]. As targeted dietary advice may be more effective than general recommendations [11, 14, 16, 24, 25], the development of strategies for change in dietary behavior on the metabotype subgroup level, especially for the ‘high-risk’ cluster 3, could improve the prevention of diabetes.

### Strengths and limitations

One of the strengths of the study is the fact that associations between diet and diabetes were investigated in a large population-based study, allowing us to perform stratified analyses by metabotype with sufficient sample sizes. Due to the lack of a uniform definition of the term ‘metabotype’, metabotyping was performed in KORA FF4 analogous to Riedl et al. [23] in KORA F4 to get comprehensive metabotypes based on a broad range of parameters [22]. However, only 16 of the 34 biochemical and anthropometric parameters originally used in F4, were available in FF4. Repeating the identification of metabotypes in F4 with the reduced set of 16 parameters also available in FF4 and comparing to the originally identified metabotypes in F4 based on 34 parameters, revealed a similar allocation of individuals to the clusters (1513 of 1729 individuals or 87.5% of participants). In addition, the newly defined metabotypes in FF4 based on the 16 parameters showed a good distinction of demographic and metabolic characteristics. Consequently, we assume to have identified metabotypes in KORA FF4 that are still comprehensive despite the reduced set of biochemical markers and we assume that these metabotypes were appropriate to consider metabolic differences in diet–diabetes associations. Another strength is the availability of extensive dietary data assessed by food frequency questionnaire and up to three 24-h food lists, which enabled the investigation of a large number of food items and their association with diabetes. However, as with all dietary assessment methods, misreporting cannot be ruled out. Further strengths include the assessment of diabetes by either a physician-validated diagnosis or an OGTT, and the availability of a large number of confounders for adjustment. Limitations of the study include the fact that a large proportion of the original S4 participants (1982 of 4261 individuals) did not participate again in the second follow-up KORA FF4 study due to death, refusal and loss to follow-up, what could have biased our results. Furthermore, due to the cross-sectional study design, no causal relationships between dietary factors and diabetes could be established and longitudinal or intervention studies considering metabolic differences are needed. In addition, samples sizes, dietary intake amounts and the diabetes prevalence varied considerably between metabotype subgroups, which could have influenced our results.

## Conclusions

Our cross-sectional results show differences in associations with diabetes for intake of fruits, total meat, processed meat, and especially for intake of SSB between distinct metabotype subgroups. This suggests an influence of metabolic characteristics on diet–diabetes associations, which may help to explain the inconsistent results of previous studies. Further, prospective and intervention studies are needed to further elucidate the causal relationships between diet and diabetes within specific metabolic subgroups. These results may enable the development of targeted dietary recommendations on the metabotype subgroup level in diabetes prevention.

## Electronic supplementary material

Below is the link to the electronic supplementary material.

Supplementary material 1 (DOC 186 kb)
