# Supplementary material for: Modifying effect of metabotype on diet–diabetes associations
Source: Eur J Nutr. 2019 May 14;59(4):1357–69. doi: 10.1007/s00394-019-01988-5 (PMC7230059; doi:10.1007/s00394-019-01988-5)
Supplement: Supplementary file 1 — Supplementary material 1 (DOC 186 kb) [file 394_2019_1988_MOESM1_ESM.doc]

# **Supplementary Material**

# **Modifying effect of metabotype on diet-diabetes associations**

**Anna Riedl^1,2,3^*, Nina Wawro, Christian Gieger, Christa Meisinger, Annette Peters**, **Wolfgang Rathmann**, **Wolfgang Koenig**, **Konstantin Strauch, Anne S. Quante, Barbara Thorand, Cornelia Huth, Hannelore Daniel, Hans Hauner, Jakob Linseisen**

^1^ Independent Research Group Clinical Epidemiology, Helmholtz Zentrum München, German Research Center for Environmental Health (GmbH), Ingolstädter Landstr. 1, 85764 Neuherberg, Germany

^2^ Chair of Epidemiology, Ludwig-Maximilians-Universität München, at UNIKA-T (Universitäres Zentrum für Gesundheitswissenschaften am Klinikum Augsburg), Neusässer Str. 47, 86156 Augsburg, Germany

^3^ German Center for Diabetes Research (DZD e.V.), Ingolstädter Landstr. 1, 85764 Neuherberg, Germany

*** Corresponding author:**

**Mailing address:** Helmholtz Zentrum München, German Research Center for Environmental Health (GmbH), Ingolstädter Landstr. 1, 85764 Neuherberg, Germany

**Email address:** anna.riedl@helmholtz-muenchen.de

**Telephone number:** +49-89-3187-3987

**Fax number:** +49-89-3187-2951

**Supplemental Table 1:** Median baseline values of anthropometric and biochemical parameters of the total study population and across the three clusters, KORA FF4 study

|  | **Total** | **Metabotypes** | | | |  |
| --- | --- | --- | --- | --- | --- | --- |
|  |  | **Cluster 1** | **Cluster 2** | | **Cluster 3** |  |
|  | **N=2218** | **N=993** | **N=779** | | **N=446** | **p-value** |
| **Clustering variables** |  |  | |  |  |  |
| BMI (kg/m²) | 27.0 (24.4, 30.5) | 24.8 (22.8, 27.1)^a^ | | 27.8 (25.7, 30.6)^b^ | **31.5** (28.5, 35.5)^c^ | **<0.0001** |
| Glucose (mg/dL) | 98 (91, 107) | 93 (88, 100)^a^ | | 99 (93, 106)^b^ | **113** (102, 134)^c^ | **<0.0001** |
| Total cholesterol/High density lipoprotein cholesterol | 3.35 (2.72, 4.13) | 2.71 (2.32, 3.09)^a^ | | **4.14** (3.67, 4.88)^b^ | 3.72 (3.13, 4.47)^c^ | **<0.0001** |
| Total cholesterol (mmol/L) | 5.56 (4.88, 6.23) | 5.25 (4.68, 5.79)^a^ | | **6.20** (5.74, 6.87)^b^ | 5.01 (4.42, 5.61)^c^ | **<0.0001** |
| Glycated hemoglobin (%) | 5.4 (5.2, 5.7) | 5.3 (5.1, 5.5)^a^ | | 5.4 (5.3, 5.7)^b^ | **5.8** (5.5, 6.5)^c^ | **<0.0001** |
| High density lipoprotein cholesterol (mmol/L) | 1.63 (1.34, 2.02) | **1.96** (1.65, 2.29)^a^ | | 1.47 (1.27, 1.71)^b^ | 1.34 (1.11, 1.58)^c^ | **<0.0001** |
| Uric acid (µmol/L) | 330 (273, 397) | 288 (246, 338)^a^ | | 356 (302, 409)^b^ | **403** (344, 467)^c^ | **<0.0001** |
| Low density lipoprotein cholesterol (mmol/L) | 3.44 (2.84, 4.06) | 3.05 (2.58, 3.49)^a^ | | **4.21** (3.79, 4.68)^b^ | 3.02 (2.56, 3.66)^a^ | **<0.0001** |
| Triglycerides (mmol/L) | 1.19 (0.88, 1.63) | 0.89 (0.70, 1.12)^a^ | | 1.49 (1.17, 2.02)^b^ | **1.55** (1.19, 2.21)^b^ | **<0.0001** |
| Leukocytes (/nL) | 5.7 (4.9, 6.8) | 5.3 (4.5, 6.2)^a^ | | 5.8 (5.0, 6.8)^b^ | **6.7** (5.8, 7.7)^c^ | **<0.0001** |
| Glutamate-pyruvate transaminase (µkat/L) | 0.38 (0.30, 0.52) | 0.33 (0.27, 0.40)^a^ | | 0.40 (0.32, 0.53)^b^ | **0.58** (0.40, 0.78)^c^ | **<0.0001** |
| Glutamate-oxaloacetate transaminase (µkat/L) | 0.38 (0.32, 0.47) | 0.35 (0.30, 0.42)^a^ | | 0.38 (0.32, 0.47)^b^ | **0.47** (0.37, 0.62)^c^ | **<0.0001** |
| Gamma-glutamyltransferase (µkat/L) | 0.41 (0.27, 0.64) | 0.32 (0.23, 0.45)^a^ | | 0.45 (0.31, 0.65)^b^ | **0.70** (0.45, 1.20)^c^ | **<0.0001** |
| Alkaline phosphatase (µkat/L) | 1.09 (0.90, 1.29) | 0.99 (0.82, 1.17)^a^ | | 1.14 (0.97, 1.30)^b^ | **1.24** (1.02, 1.49)^c^ | **<0.0001** |
| High-sensitive C-reactive protein (mg/L) | 1.21 (0.58, 2.60) | 0.80 (0.42, 1.60)^a^ | | 1.36 (0.70, 2.65)^b^ | **2.63** (1.22, 6.19)^c^ | **<0.0001** |
| Insulin (mU/mL) | 9.2 (6.3, 13.5) | 6.8 (5.0, 9.4)^a^ | | 10.0 (7.4, 13.5)^b^ | **17.0** (12.0, 23.7)^c^ | **<0.0001** |
| Median (25^th^, 75^th^ percentile) calculated by the means of medians, 25^th^ and 75^th^ percentiles over all five imputed datasets; Kruskal-Wallis test (and Kruskal-Wallis post-hoc test with Bonferroni correction) was calculated for each of the five imputed datasets and the p-value was determined from the mean of the Kruskal-Wallis test statistics over the five imputed datasets.  Significant results (p<0.05) are **highlighted**. Different superscript letters between clusters indicate a significant difference between clusters, whereas the same superscript letters between clusters indicate no significant difference between clusters.  Underlined values represent the lowest value across the clusters; the **bold** values represent the highest.  BMI, body mass index; KORA, Cooperative Health Research in the Region of Augsburg. | | | | | | |

**KORA FF4 study population with information on biochemical and anthropometric parameters**

n=2279, v=16

**Exclusion criteria:**

- Fasting <8 h (n=54) before blood collection
- Variables with >20% missing values (n=0)
- Subjects with >10% missing values (n=7)

**Identification of metabotypes** by k-means cluster analysis based on 16 biochemical and anthropometric parameters

n=2218, v=16

**Exclusion criteria:**

- Subjects with type 1 diabetes mellitus (n=4)
- Subjects with unclear glucose tolerance status (n=67)
- Subjects with missing dietary intake data (n=628)
- Subjects with missing covariate data (n=2)

**Analysis of diet-diabetes associations in the total study population and stratified by metabotype** using logistic regression models

Basic model: adjusted for age, sex, energy intake

Fully adjusted model: additionally adjusted for waist circumference, family history of diabetes, physical activity, smoking, education, hypertension, metabotype

Likelihood ratio tests to investigate interactions between diet and metabotypes

n=1517

**Supplemental Fig. 1:** Overview of the analysis approach, KORA FF4 study

KORA, Cooperative Health Research in the Region of Augsburg

**Supplemental Table 2:** Age-, sex- and energy intake-adjusted associations between the consumption of various food items and nutrients with UDM/Prevalent T2DM in the total study population, KORA FF4 study

|  | **Basic model** | |
| --- | --- | --- |
| Food or Nutrient | OR | 95% CI |
| Fruits (50 g/d) | **0.88** | **0.78 - 0.99** |
| Vegetables (50 g/d) | 0.93 | 0.77 - 1.10 |
| Potatoes (50 g/d) | 0.99 | 0.65 - 1.47 |
| Total meat (50 g/d) | **2.53** | **1.98 - 3.28** |
| Red meat (50 g/d) | **2.33** | **1.08 - 4.89** |
| Poultry (50 g/d) | 2.08 | 0.64 - 6.22 |
| Processed meat (50 g/d) | **3.50** | **2.51 - 4.93** |
| Eggs (50 g/d) | 1.18 | 0.61 - 2.20 |
| Total dairy (50 g/d) | 0.97 | 0.89 - 1.06 |
| Milk (50 g/d) | 0.94 | 0.83 - 1.04 |
| Yogurt (50 g/d) | 1.06 | 0.87 - 1.27 |
| Cheese (50 g/d) | 1.43 | 0.76 - 2.67 |
| Coffee (50 g/d) | 0.99 | 0.94 - 1.05 |
| Fruit and vegetable juice (50 g/d) | 1.01 | 0.92 - 1.11 |
| SSB (50 g/d) | **1.12** | **1.05 - 1.19** |
| Moderate alcohol consumption^a^ | **0.67** | **0.46 - 0.96** |
| High alcohol consumption^a^ | 0.63 | 0.39 - 1.00 |
| Total fiber (10 g/d) | **0.46** | **0.28 - 0.75** |
| Logistic regression models: reference category=NGT/prediabetes. Basic models adjusted for age, sex and energy intake. Significant results (p<0.05) printed in **bold**.  N=1517.  ^a^ Compared against low alcohol intake (<5 g/d for men, <2 g/d for women as reference category); moderate considered 5 to <20 g/d for men, 2 to <10 g/d for women; high considered ≥20 g/d for men, ≥10 g/d for women.  CI, confidence interval; KORA, Cooperative Health Research in the Region of Augsburg; NGT, normal glucose tolerance; OR, odds ratio; SSB, sugar sweetened beverages; T2DM, type 2 diabetes mellitus; UDM, undetected diabetes mellitus. | | |

**Supplemental Table 3:** Age-, sex- and energy intake-adjusted associations between the consumption of various food items and nutrients with UDM/Prevalent T2DM stratified by metabotype subgroup, KORA FF4 study

|  | **Cluster 1/Cluster 2**  N=1217 | | **Cluster 3**  N=300 | | |
| --- | --- | --- | --- | --- | --- |
| Food or Nutrient | OR | 95% CI | OR | 95% CI |  |
| Fruits (50 g/d) | 0.90 | 0.74 - 1.08 | 0.85 | 0.72 - 1.01 | |
| Vegetables (50 g/d) | 1.07 | 0.82 - 1.37 | 1.01 | 0.77 - 1.32 | |
| Potatoes (50 g/d) | 0.83 | 0.43 - 1.54 | 1.42 | 0.75 - 2.73 | |
| Total meat (50 g/d) | **1.99** | **1.33 - 2.99** | **1.52** | **1.06 - 2.20** | |
| Red meat (50 g/d) | 1.40 | 0.35 - 4.93 | 1.48 | 0.50 - 4.43 | |
| Poultry (50 g/d) | 0.93 | 0.10 - 6.57 | 1.68 | 0.34 - 8.35 | |
| Processed meat (50 g/d) | **2.69** | **1.58 - 4.62** | **1.75** | **1.10 - 2.82** | |
| Eggs (50 g/d) | 1.70 | 0.66 - 4.03 | 0.43 | 0.15 - 1.20 | |
| Total dairy (50 g/d) | 0.97 | 0.84 - 1.11 | 1.02 | 0.90 - 1.17 | |
| Milk (50 g/d) | 0.95 | 0.79 - 1.12 | 0.99 | 0.83 - 1.18 | |
| Yogurt (50 g/d) | 1.01 | 0.73 - 1.35 | 1.16 | 0.87 - 1.58 | |
| Cheese (50 g/d) | 1.90 | 0.71 - 4.89 | 0.93 | 0.35 - 2.43 | |
| Coffee (50 g/d) | 0.97 | 0.89 - 1.07 | 1.07 | 0.99 - 1.17 | |
| Fruit and vegetable juice (50 g/d) | 1.00 | 0.85 - 1.16 | 0.96 | 0.83 - 1.09 | |
| SSB (50 g/d) | 0.94 | 0.75 - 1.10 | **1.16** | **1.06 - 1.27** | |
| Moderate alcohol consumption^a^ | 0.76 | 0.43 - 1.35 | 0.88 | 0.49 - 1.57 | |
| High alcohol consumption^a^ | 0.77 | 0.37 - 1.58 | 0.73 | 0.36 - 1.50 | |
| Total fiber (10 g/d) | 0.77 | 0.37 - 1.56 | 0.84 | 0.38 - 1.84 | |
| Logistic regression models: reference category=NGT/prediabetes. Basic models adjusted for age, sex and energy intake. Significant results (p<0.05) printed in **bold**.  N=1517.  ^a^ Compared against low alcohol intake (<5 g/d for men, <2 g/d for women as reference category); moderate considered 5 to <20 g/d for men, 2 to <10 g/d for women; high considered ≥20 g/d for men, ≥10 g/d for women.  CI, confidence interval; KORA, Cooperative Health Research in the Region of Augsburg; NGT, normal glucose tolerance; OR, odds ratio; SSB, sugar sweetened beverages; T2DM, type 2 diabetes mellitus; UDM, undetected diabetes mellitus. | | | | | |

**Supplemental Table 4:** Intermediate adjusted associations (without adjustment for hypertension and waist circumference) between the consumption of various food items and nutrients with UDM/Prevalent T2DM in the total study population, KORA FF4 study

|  | **Intermediate adjusted model** | |
| --- | --- | --- |
| Food or Nutrient | OR | 95% CI |
| Fruits (50 g/d) | **0.86** | **0.75 - 0.98** |
| Vegetables (50 g/d) | 1.13 | 0.92 - 1.37 |
| Potatoes (50 g/d) | 1.08 | 0.68 - 1.71 |
| Total meat (50 g/d) | **1.71** | **1.27 - 2.30** |
| Red meat (50 g/d) | 1.21 | 0.47 - 3.02 |
| Poultry (50 g/d) | 1.52 | 0.39 - 5.62 |
| Processed meat (50 g/d) | **2.10** | **1.45 - 3.08** |
| Eggs (50 g/d) | 0.97 | 0.46 - 1.98 |
| Total dairy (50 g/d) | 0.99 | 0.90 - 1.10 |
| Milk (50 g/d) | 0.95 | 0.83 - 1.07 |
| Yogurt (50 g/d) | 1.08 | 0.87 - 1.33 |
| Cheese (50 g/d) | 1.60 | 0.77 - 3.32 |
| Coffee (50 g/d) | 1.02 | 0.96 - 1.09 |
| Fruit and vegetable juice (50 g/d) | 0.97 | 0.88 - 1.08 |
| SSB (50 g/d) | **1.10** | **1.03 - 1.18** |
| Moderate alcohol consumption^a^ | 0.90 | 0.60 - 1.37 |
| High alcohol consumption^a^ | 0.83 | 0.48 - 1.41 |
| Total fiber (10 g/d) | 0.95 | 0.54 - 1.64 |
| Logistic regression models: reference category=NGT/prediabetes. Intermediate adjusted models adjusted for age, sex, energy intake, family history of diabetes, physical activity, smoking, education, and metabotype. Significant results (p<0.05) printed in **bold**.  N=1517.  ^a^ Compared against low alcohol intake (<5 g/d for men, <2 g/d for women as reference category); moderate considered 5 to <20 g/d for men, 2 to <10 g/d for women; high considered ≥20 g/d for men, ≥10 g/d for women.  CI, confidence interval; KORA, Cooperative Health Research in the Region of Augsburg; NGT, normal glucose tolerance; OR, odds ratio; SSB, sugar sweetened beverages; T2DM, type 2 diabetes mellitus; UDM, undetected diabetes mellitus. | | |

**Supplemental Table 5:** Intermediate adjusted associations (without adjustment for hypertension and waist circumference) between the consumption of various food items and nutrients with UDM/Prevalent T2DM stratified by metabotype subgroup, KORA FF4 study

|  | **Cluster 1/Cluster 2**  **N=1217** | | **Cluster 3**  **N=300** | |  |
| --- | --- | --- | --- | --- | --- |
| Food or Nutrient | OR | 95% CI | OR | 95% CI | P-value interaction^b^ |
| Fruits (50 g/d) | 0.92 | 0.75 - 1.11 | **0.83** | **0.69 - 0.99** | 0.23 |
| Vegetables (50 g/d) | 1.22 | 0.92 - 1.59 | 1.05 | 0.78 - 1.41 | 0.72 |
| Potatoes (50 g/d) | 0.80 | 0.41 - 1.52 | 1.49 | 0.77 - 2.96 | 0.94 |
| Total meat (50 g/d) | **1.99** | **1.29 - 3.07** | **1.50** | **1.01 - 2.28** | 0.16 |
| Red meat (50 g/d) | 1.20 | 0.26 - 4.83 | 1.06 | 0.32 - 3.49 | 0.43 |
| Poultry (50 g/d) | 1.02 | 0.09 - 8.36 | 2.34 | 0.41 - 13.81 | 0.64 |
| Processed meat (50 g/d) | **2.66** | **1.54 - 4.68** | **1.73** | **1.04 - 2.95** | 0.11 |
| Eggs (50 g/d) | 2.04 | 0.74 - 5.26 | 0.42 | 0.14 - 1.20 | **0.02** |
| Total dairy (50 g/d) | 0.96 | 0.83 - 1.11 | 1.03 | 0.89 - 1.18 | 0.59 |
| Milk (50 g/d) | 0.91 | 0.75 - 1.08 | 0.98 | 0.82 - 1.17 | 0.64 |
| Yogurt (50 g/d) | 1.03 | 0.74 - 1.37 | 1.20 | 0.88 - 1.67 | 0.43 |
| Cheese (50 g/d) | 2.72 | 0.98 - 7.35 | 1.05 | 0.38 - 2.96 | 0.13 |
| Coffee (50 g/d) | 0.97 | 0.88 - 1.06 | 1.08 | 0.98 - 1.18 | 0.09 |
| Fruit and vegetable juice (50 g/d) | 1.00 | 0.85 - 1.15 | 0.96 | 0.84 - 1.11 | 0.50 |
| SSB (50 g/d) | 0.93 | 0.74 - 1.08 | **1.20** | **1.09 - 1.33** | **0.01** |
| Moderate alcohol consumption^a^ | 0.89 | 0.49 - 1.61 | 0.96 | 0.52 - 1.77 | 0.76 |
| High alcohol consumption^a^ | 0.86 | 0.40 - 1.80 | 0.76 | 0.35 - 1.61 |
| Total fiber (10 g/d) | 1.09 | 0.50 - 2.32 | 0.86 | 0.36 - 2.02 | 0.26 |
| Logistic regression models: reference category=NGT/prediabetes. Intermediate adjusted models adjusted for age, sex, energy intake, family history of diabetes, physical activity, smoking, and education. Significant results (p<0.05) printed in **bold**.  N=1517.  ^a^ Compared against low alcohol intake (<5 g/d for men, <2 g/d for women as reference category); moderate considered 5 to <20 g/d for men, 2 to <10 g/d for women; high considered ≥20 g/d for men, ≥10 g/d for women.  ^b^ P-value of likelihood ratio test for the comparison of models with and without the interaction term of metabotype and the respective food or nutrient.  CI, confidence interval; KORA, Cooperative Health Research in the Region of Augsburg; NGT, normal glucose tolerance; OR, odds ratio; SSB, sugar sweetened beverages; T2DM, type 2 diabetes mellitus; UDM, undetected diabetes mellitus. | | | | | |
